# Electrocardiographic characteristics associated with late gadolinium enhancement and prognostic value in patients with dilated cardiomyopathy

**DOI:** 10.3389/fcvm.2023.1281563

**Published:** 2023-10-18

**Authors:** Punyanuch Chayanopparat, Thananya Boonyasirinant, Natthaporn Prapan, Supamongkol Phoopattana, Yodying Kaolawanich

**Affiliations:** Division of Cardiology, Department of Medicine, Faculty of Medicine Siriraj Hospital, Mahidol University, Bangkok, Thailand

**Keywords:** cardiac magnetic resonance, dilated cardiomyopathy, electrocardiography, late gadolinium enhancement, prognostic value, sudden cardiac death

## Abstract

**Background:**

Late gadolinium enhancement (LGE) cardiac magnetic resonance (CMR) imaging has emerged as an important tool for assessment of patients with dilated cardiomyopathy (DCM). Electrocardiography (ECG) is an accessible, reproducible, low-cost diagnostic and prognostic tool. This study aimed to investigate the ECG characteristics associated with LGE, as well as to assess the prognostic significance of ECG in patients with DCM.

**Methods:**

Consecutive patients diagnosed with DCM by CMR [left ventricular ejection fraction (LVEF) < 50%] between 2011 and 2020 were included. Multivariable analysis was conducted to evaluate ECG predictors associated with LGE. Receiver operating characteristic (ROC) analysis was performed to assess the diagnostic performance of ECG in combination of clinical data and LVEF for LGE. Two composite outcomes were also assessed among patients with and without ECG predictors: (1) sudden cardiac death (SCD), sustained ventricular arrhythmia, or appropriate implantable cardioverter-defibrillator (ICD) therapy, and (2) all-cause death or hospitalization for heart failure.

**Results:**

A total of 422 patients, with a mean age of 59.5 ± 16.3 years (58.3% male), were included. LGE was present in 169 (40%) of the patients. Multivariable analysis identified lateral inverted T-waves, intraventricular conduction delay, low voltage, and fragmented QRS as independent predictors of LGE. ROC analysis showed a significant increase in the area under the curve (AUC) when ECG predictors of the four aforementioned characteristics were added to the clinical-LVEF model (AUC 0.66, 95% CI 0.59–0.71 vs. 0.72, 95% CI 0.67–0.78, *p* = 0.003). During a median follow-up of 2.7 years (IQR 0.8, 5.2), 16 events of SCD, sustained ventricular arrhythmia, or appropriate ICD therapy, and 70 events of all-cause death or hospitalization for heart failure occurred. ECG predictors were independently associated with SCD, sustained ventricular arrhythmia, or appropriate ICD therapy (HR 4.84, 95% CI 1.34–17.40, *p* = 0.01). However, ECG predictors were not associated with all-cause death or hospitalization for heart failure (HR 1.22, 95% CI 0.76–1.96, *p* = 0.39).

**Conclusion:**

In patients with DCM, lateral inverted T-waves, intraventricular conduction delay, low voltage, and fragmented QRS were independently associated with LGE. Additionally, these ECG predictors had prognostic value for predicting SCD, sustained ventricular arrhythmia, or appropriate ICD therapy, assisting clinicians in stratifying SCD risk and identifying primary prevention ICD implantation candidates.

## Introduction

Non-ischemic dilated cardiomyopathy (DCM) is a heterogeneous group of conditions, mostly caused by a genetic predisposition, consisting in left ventricular (LV) dilatation and dysfunction. DCM is an important cause of sudden cardiac death (SCD) and heart failure. Several previous studies have demonstrated the prognostic value of late gadolinium enhancement (LGE) with cardiac magnetic resonance (CMR) in patients with DCM (1–4). A meta-analysis by Becker et al., including 4,554 patients with DCM, demonstrated that LGE substantially worsens the prognosis for adverse cardiovascular (CV) events in DCM patients, and its absence indicates LV reverse remodeling (4).

The most recent European Society of Cardiology (ESC) guidelines have recommended that CMR with LGE should be considered in patients with DCM for assessing the etiology and the risk of SCD (5). However, in developing countries, the ability to send all DCM patients for CMR is limited. Electrocardiography (ECG) is an accessible, reproducible, low-cost diagnostic and prognostic tool. Previous studies have reported that ECG findings such as low QRS amplitude, anterolateral inverted T-waves, and fragmented QRS complex carry a heightened risk for major ventricular arrhythmias and adverse cardiac events (6–9).

Despite the potential of several ECG features to indicate a susceptibility to life-threatening cardiac arrhythmias, there is a lack of data regarding specific ECG characteristics associated with LGE. Additionally, none of these ECG characteristics are incorporated into the recommendations for primary prevention implantation of implantable cardioverter-defibrillators (ICDs), which currently rely solely on left ventricular ejection fraction (LVEF) and the New York Heart Association (NYHA) class (5).

In this study, our objective was to evaluate specific ECG characteristics associated with LGE in patients with DCM undergoing CMR, along with ECG predictors for adverse events. The results could enhance the risk stratification for DCM patients, assisting in the selection of candidates for CMR and future ICD implantation.

## Methods

### Study population

**Figure 1:**
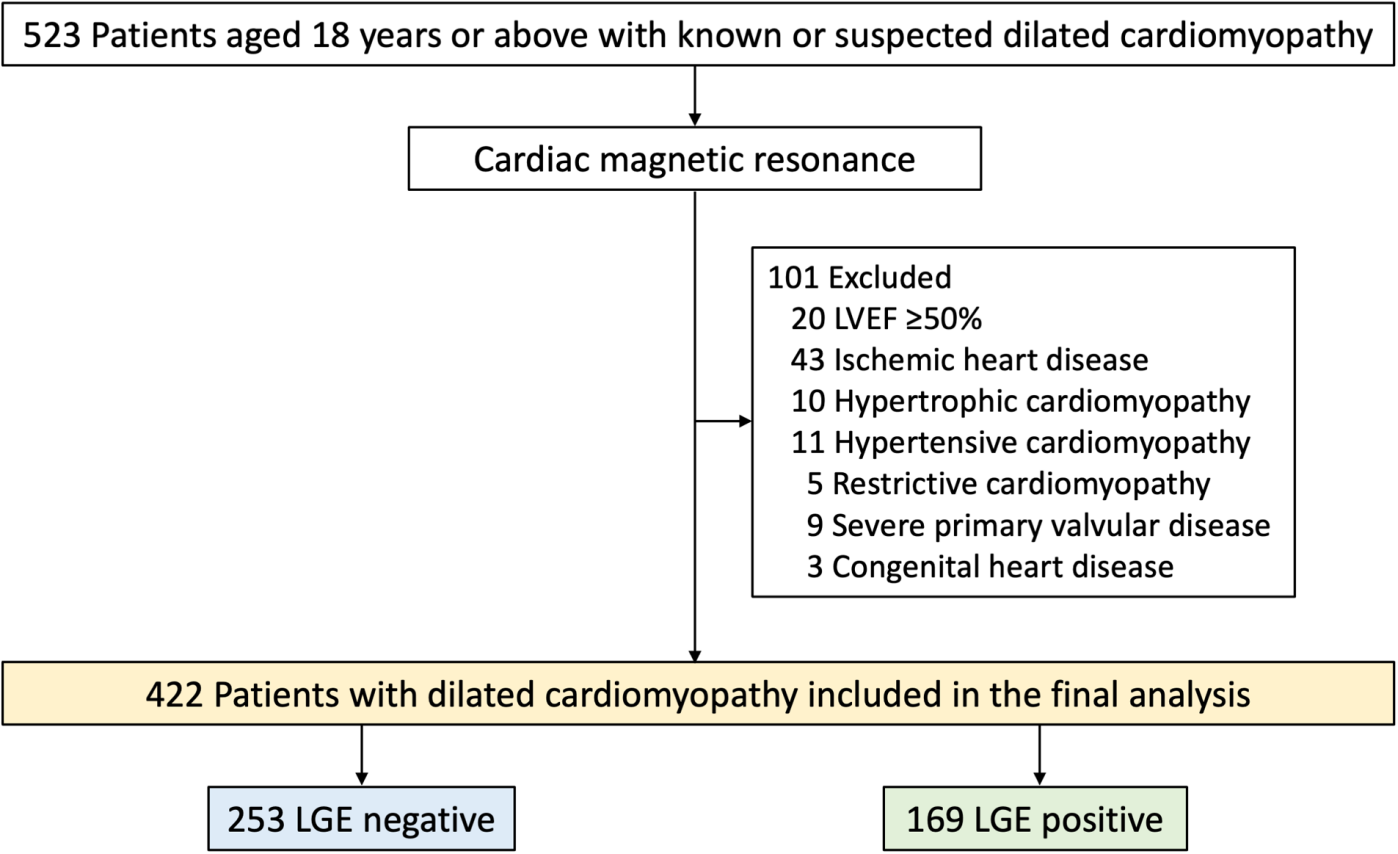
Flow chart of study population. LVEF, left ventricular ejection fraction; LGE, late gadolinium enhancement.

Consecutive patients aged 18 years or above with known or suspected DCM referred for CMR in a tertiary center in Thailand during 2010 and 2020 were studied. DCM was defined in accordance with the criteria of the World Health Organization (10). Patients were excluded if they had severe primary valvular disease, a diagnosis of other nonischemic disorders such as hypertrophic or infiltrative cardiomyopathy, or congenital heart disease. Additionally, those with CMR-confirmed subendocardial or transmural LV scarring indicative of previous myocardial infarction, a positive pharmacologic stress test indicating myocardial ischemia, or an LVEF ≥ 50%, were also excluded (Figure 1). The study was done in accordance with the Declaration of Helsinki. The institutional ethics committee [Siriraj Institutional Review Board (SIRB), Faculty of Medicine Siriraj Hospital, Mahidol University] approved this retrospective study and waived the need for additional written informed consent.

### Electrocardiographic analysis

A twelve-lead ECG (GE MAC 1200; GE Marquette Medical Systems, Milwaukee, WI, USA) was obtained on the CMR date as part of the routine CMR protocol. Each ECG was reviewed by two trained cardiologists who were blinded to the patients’ data, and discordant results were resolved by a senior cardiologist. The ECGs were coded according to the Minnesota Code. ECG measurements included PR, RR, QT, and QRS intervals. The presence of a left bundle branch block (LBBB), complete right bundle branch block (RBBB), or interventricular conduction delay (IVCD) was defined according to the Minnesota Code. A premature ventricular contraction (PVC) recorded on a 12-lead ECG is characterized by a wide and atypical QRS complex, occurring earlier than expected in the regular heart rhythm and not preceded by a premature *P* wave. A fragmented QRS complex was defined based on a published article (11).

### CMR protocol and image analysis

CMR was performed on 1.5-T or 3.0-T systems. (Philips Medical Systems, Best, the Netherlands) A standardized protocol as described by the Society for Cardiovascular Magnetic Resonance for the assessment of LV structure and function and myocardial scarring with cine and LGE imaging, respectively, was used (12). In brief, cine imaging was performed using a segmented steady-state free precession (SSFP) sequence with short-axis images acquired throughout the entire left ventricle. Long-axis images were obtained in standard 2-, 3-, and 4-chamber orientations. LGE imaging was performed 10 min after 0.15 mmol/kg gadolinium contrast administration using a standard 3D segmented inversion-recovery sequence and images were obtained in short- and long-axis locations matching those of cine imaging. Typical CMR parameters for 1.5-T for cine images included 8 mm slice thickness, 70-degree flip angle, repetition time (TR)/echo time (TE)/number of excitations = 3.7/1.8/2, 390 × 312 mm field of view, 256 × 240 matrix, and 1.52 × 1.3 reconstruction pixel. The parameters for LGE images were 3D segmented-gradient-echo inversion- recovery sequence with 8 mm slice thickness, 15-degree flip angle, 1.5 SENSitivity Encoding (SENSE) factor, TR/TE = 4.1/1.25 ms, 303 × 384 mm field of view, 240 × 256 matrix, and 1.26 × 1.5 mm reconstruction pixel.

Standard LV volumes, mass, and EF were quantitatively measured from the stack of short-axis SSFP cine images. LVEF was calculated from left ventricular end-systolic (LVESV) and end-diastolic volume (LVEDV) data. LV mass was calculated from the summation of subtraction of left ventricular end-diastolic epicardial and endocardial area and slice thickness of each slide. LGE images were analyzed using visual assessment. LGE was considered present only if confirmed on both the short-axis and at least one other orthogonal plane (13). Midwall LGE was only considered present if the area of LGE was confined to the intermural (3). Subepicardial LGE was only considered present if the area of LGE was confined to subepicardial layers (3), and isolated LGE was defined as focal LGE (14).

### Clinical follow-up

Follow-up data were collected from clinical visits and medical records. Clinical event adjudication was conducted while maintaining complete blindness to clinical, ECG, and CMR data. Patients were followed for two composite outcomes, which included: (1) SCD, sustained ventricular arrhythmia, or appropriate ICD therapy; and (2) all-cause death or hospitalization for heart failure (15).

### Statistical analysis

All statistical analyses were performed using SPSS Statistics for Windows version 20.0 (SPSS, Inc., Chicago, IL, USA). Continuous variables with normal distribution were presented as mean ± standard deviation, and continuous variables with non-normal distribution were presented as median and interquartile ranges (IQR). The normality of the distribution of variables was examined by the Kolmogorov-Smirnov test. Categorical variables were present as absolute numbers and percentages. Differences between patients with and without LGE in terms of baseline, ECG and image characteristics were compared using the Student’s unpaired *t*-test or the Mann-Whitney *U* test for continuous variables, while the chi-square test or Fisher’s exact test was used for categorical variables, as appropriate.

Binary logistic regression analysis was performed to identify significant predictors of LGE from baseline and ECG characteristics. Variables with a *p*-value < 0.05 from univariable analysis were entered into multivariable analysis. The results of the univariable and multivariable analyses are given as odds ratios (OR) along with their respective 95% confidence intervals (CI). A *p*-value less than 0.05 was considered statistically significant for all tests. We performed receiver operating characteristic (ROC) curve analysis to assess the diagnostic performance of clinical data alone and when combined with ECG to predict LGE. A comparison of the area under the curve (AUC) was made for these two approaches.

We assessed two composite outcomes: (1) SCD, sustained ventricular arrhythmia, or appropriate ICD therapy; and (2) all-cause death or hospitalization for heart failure. We employed the Kaplan-Meier method to compare the rates of these composite outcomes between patients with and without ECG predictors, using the log-rank test. To analyze the predictors of composite outcomes, we performed a Cox regression analysis to assess univariable predictors. Variables with a *p*-value < 0.05 from the univariable analysis were included in the multivariable analysis.

## Results

### Study population

A total of 523 patients with known or suspected DCM were clinically referred for CMR (Figure 1). Of these, 101 were excluded and 422 were included in the final analysis. Baseline characteristics are summarized in Table 1. Mean age was 59.5 ± 16.3 years (range 18–89 years) and 58% were male. The most common symptoms of DCM included dyspnea (75.1%), syncope or palpitation (12.3%), and chest pain (7.6%). Forty-nine percent had a history of heart failure and 10% had NYHA functional class III or IV. The mean LVEF was 32.5 ± 10.7% and 169 patients (40.0%) had LGE present. Patients with LGE exhibited lower systolic blood pressure (BP), a higher prevalence of heart failure, and a greater usage of loop diuretics compared to those without LGE. Additionally, patients with LGE demonstrated significantly more severe LV dilatation and systolic dysfunction than those without LGE.

**Table 1:** Baseline clinical and CMR characteristics of the study population.

	All patients (*n* = 422)	LGE present (*n* = 169)	LGE absent (*n* = 253)	*P*-value
Age (years)	59.5 ± 16.3	58.7 ± 15.5	60.0 ± 16.7	0.41
Male, *n* (%)	246 (58.3)	105 (62.1)	141 (55.7)	0.23
Body mass index (kg/m^2^)	24.2 ± 5.3	23.8 ± 5.3	24.5 ± 5.2	0.15
Systolic blood pressure (mmHg)	130.3 ± 23.0	126.1 ± 22.2	133.1 ± 23.1	***0.002***
Diastolic blood pressure (mmHg)	75.3 ± 15.9	74.3 ± 15.3	75.9 ± 16.2	0.30
Chest pain, *n* (%)	32 (7.6)	11 (6.5)	21 (8.3)	0.58
Dyspnea, *n* (%)	317 (75.1)	135 (79.9)	182 (71.9)	0.06
Syncope or palpitation, *n* (%)	52 (12.3)	26 (15.4)	26 (10.3)	0.13
History of heart failure, *n* (%)	207 (49.1)	99 (58.6)	108 (42.7)	***0.001***
NYHA functional class, *n* (%)				0.19
I–II	380 (90)	148 (87.6)	232 (91.7)	
III–IV	42 (10)	21 (12.4)	21 (8.3)	
Medical history, *n* (%)				
Hypertension	243 (57.6)	89 (52.7)	154 (60.9)	0.11
Diabetes mellitus	136 (32.2)	56 (33.1)	80 (31.6)	0.75
Hyperlipidemia	172 (40.8)	75 (44.4)	97 (38.3)	0.22
Smoker	43 (10.2)	17 (10.0)	26 (10.3)	0.99
Alcohol excess^a^	38 (9.0)	15 (8.9)	23 (9.1)	0.99
Ischemic stroke	30 (7.1)	12 (7.1)	18 (7.1)	0.99
Sustained ventricular arrhythmia	23 (5.5)	12 (7.1)	11 (4.3)	0.28
Medications at baseline, *n* (%)				
ACE inhibitor or ARB	260 (61.6)	110 (65.1)	150 (59.3)	0.26
Anticoagulant	63 (14.9)	19 (11.2)	44 (17.4)	0.10
Antiplatelet	172 (40.8)	82 (48.5)	90 (35.6)	***0.01***
Beta-blocker	289 (68.5)	125 (73.9)	164 (64.8)	0.05
Calcium channel blocker	49 (33.4)	17 (10.1)	32 (12.6)	0.44
Digoxin	57 (13.5)	27 (15.9)	30 (11.9)	0.25
Loop diuretic	229 (54.3)	109 (64.5)	120 (47.4)	***0.001***
Spironolactone	114 (27.0)	48 (28.4)	66 (26.1)	0.66
Statin	182 (43.1)	73 (43.2)	109 (43.1)	0.99
Oral hypoglycemic drug	75 (17.8)	32 (18.9)	43 (17.0)	0.60
Insulin	7 (1.7)	5 (2.9)	2 (7.9)	0.12
CMR				
LVEDV index (ml/m^2^)	143.8 ± 40.3	154.5 ± 42.4	136.5 ± 37.2	**<*0.001***
LVESV index (ml/m^2^)	99.5 ± 39.6	112.1 ± 42.9	90.9 ± 34.8	**<*0.001***
LV mass index (g/m^2^)	74.2 ± 24.8	77.2 ± 26.9	72.1 ± 23.1	***0.04***
LV ejection fraction (%)	32.5 ± 10.7	29.1 ± 11.0	34.7 ± 9.9	**<*0.001***
LGE present, *n* (%)	169 (40.0)	169 (100)	0 (0)	**<*0.001***
Midwall pattern, *n* (%)	125 (29.6)	125 (74.0)	0 (0)	**<*0.001***
Subepicardial pattern, *n* (%)	27 (6.4)	27 (16.0)	0 (0)	**<*0.001***
Focal pattern, *n* (%)	17 (4.0)	17 (10.0)	0 (0)	**<*0.001***

ACE, angiotensin-converting enzyme; ARB, angiotensin II receptor blocker; CMR, cardiac magnetic resonance; LGE, late gadolinium enhancement; LV, left ventricular; LVEDV, left ventricular end-diastolic volume; LVESV, left ventricular end-systolic volume; NYHA, New York Heart Association.

Bold-italic *P*-value are <0.05 indicates statistical significance.

^a^ Defined as consistent intake of 4 or more units/d for men and 3 or more units/d for women.

### Electrocardiographic characteristics

Table 2 shows ECG characteristics in DCM patients, with only 10% having a completely normal ECG and 15% demonstrating atrial fibrillation or flutter. Approximately 20% exhibited left atrial enlargement or LV hypertrophy. Patients with LGE showed a significantly higher prevalence of lateral inverted T-waves (11.8% vs. 2.4%, *p* < 0.001) and anterior Q waves (9.7% vs. 3.3%, *p* = 0.001) compared to those without LGE. Additionally, patients with LGE had a higher prevalence of IVCD (10.0% vs. 3.2%, *p* = 0.005), low voltages (10.6% vs. 2.8%, *p* = 0.001), and fragmented QRS (30.8% vs. 20.9%, HR *p* = 0.02) than those without LGE. However, patients with LGE seemed to exhibit lower rates of LV hypertrophy compared to those without LGE (Sokolow-Lyon criteria: 16.5% vs. 23.7%, *p* = 0.08).

**Table 2:** Electrocardiographic characteristics of the study population.

	All patients (*n* = 422)	LGE present (*n* = 169)	LGE absent (*n* = 253)	*P*-value
Normal ECG, *n* (%)	43 (10.2)	14 (8.2)	29 (11.5)	0.33
Rhythm, *n* (%)				0.22
Sinus	358 (84.8)	148 (87.5)	210 (83.0)	
Atrial fibrillation or atrial flutter	64 (15.2)	21 (12.5)	43 (17.0)	
Heart rate (beats per minute)	83.9 ± 20.1	83.6 ± 20.7	84.0 ± 19.8	0.84
QRS axis (°)	14.2 ± 56.5	13.7 ± 63.0	14.5 ± 51.8	0.88
Left axis deviation, *n* (%)	92 (21.8)	45 (26.6)	47 (18.5)	0.06
Right axis deviation, *n* (%)	25 (5.9)	11 (6.5)	14 (5.5)	0.68
PR interval (ms)	176.4 ± 34.7	179.0 ± 32.4	174.4 ± 36.2	0.23
QRS duration (ms)	110.9 ± 27.8	114.0 ± 28.8	108.9 ± 26.9	0.06
QRS complex widening (>120 ms), *n* (%)	113 (26.8)	52 (30.8)	61 (24.1)	0.14
QT interval (ms)	406.7 ± 55.2	408.2 ± 59.9	405.4 ± 51.8	0.57
Left atrial enlargement, *n* (%)	91 (21.6)	40 (23.7)	51 (20.1)	0.40
Right atrial enlargement, *n* (%)	12 (2.8)	5 (3.0)	7 (2.8)	0.99
Left ventricular hypertrophy, *n* (%)				
Sokolow-Lyon	88 (20.9)	28 (16.5)	60 (23.7)	0.08
Cornell	67 (15.9)	27 (16.0)	40 (15.8)	0.99
Right ventricular hypertrophy, *n* (%)	7 (1.7)	2 (1.2)	5 (2.0)	0.71
Inverted T-waves, *n* (%)^a^				
Anterior leads	16 (4.5)	10 (6.9)	6 (2.9)	0.07
Inferior leads	4 (1.1)	3 (2.1)	1 (0.5)	0.17
Lateral leads	22 (6.2)	17 (11.8)	5 (2.4)	<***0.001***
ST depression, *n* (%)^a^				
Anterior leads	11 (3.1)	6 (4.1)	5 (2.4)	0.36
Inferior leads	8 (2.3)	5 (3.4)	3 (1.4)	0.21
Lateral leads	33 (9.3)	17 (11.8)	16 (7.7)	0.19
Q waves, *n* (%)^a^				
Anterior leads	21 (5.9)	14 (9.7)	7 (3.3)	***0.01***
Inferior leads	11 (3.1)	7 (4.9)	4 (1.9)	0.11
Lateral leads	11 (3.1)	6 (4.2)	5 (2.4)	0.34
First-degree atrioventricular block, *n* (%)	37 (8.8)	20 (11.8)	17 (6.7)	0.08
Premature ventricular complex, *n* (%)	65 (15.4)	30 (17.7)	35 (13.8)	0.27
Left bundle branch block, *n* (%)	65 (15.4)	24 (14.2)	41 (16.2)	0.58
Complete right bundle branch block, *n* (%)	23 (5.5)	11 (6.5)	12 (4.7)	0.42
Intraventricular conduction delay, *n* (%)	25 (5.9)	17 (10.0)	8 (3.2)	***0.005***
Low voltages, *n* (%)	25 (5.9)	18 (10.6)	7 (2.8)	***0.001***
Fragmented QRS, *n* (%)	105 (24.9)	52 (30.8)	53 (20.9)	***0.02***
Wolff-Parkinson-White syndrome, *n* (%)	4 (0.9)	1 (0.6)	3 (1.2)	0.65

ECG, electrocardiography; LGE, late gadolinium enhancement.

Bold-italic *P*-value are <0.05 indicates statistical significance.

^a^ Data regarding inverted T-waves, ST depression, and Q waves are available in 144 patients with LGE and 209 patients without LGE (excluding patients with left bundle branch block and Wolff-Parkinson-White syndrome).

### Clinical and electrocardiographic predictors of LGE

Supplementary Table S1 presents the results of the univariable Cox regression analysis of clinical and ECG predictors associated with LGE. Systolic BP, history of heart failure, lateral inverted T-waves, IVCD, low voltages, and fragmented QRS were found to be associated with LGE in the univariable analysis. Table 3 demonstrates the multivariable Cox regression analyses of ECG predictors associated with LGE. Four ECG characteristics, including lateral inverted T-waves (OR 5.80, 95% CI 2.02–16.65, *p* = 0.001), IVCD (OR 4.99, 95% CI 1.86–13.39, *p* = 0.001), low voltages (OR 3.60, 95% CI 1.69–7.67, *p* = 0.001), and fragmented QRS (OR 2.77, 95% CI 1.41–5.46, *p* = 0.003), were independently associated with LGE.

**Table 3:** Univariable and multivariable Cox regression analyses of electrocardiographic predictors associated with LGE.

	Univariable analysis		Multivariable analysis	
	Odd ratio (95% CI)	*P*-value	Odd ratio (95% CI)	*P*-value
Lateral inverted T-waves	5.46 (1.97, 15.17)	***0.001***	5.80 (2.02, 16.65)	***0.001***
Intraventricular conduction delay	3.42 (1.44, 8.13)	***0.005***	4.99 (1.86, 13.39)	***0.001***
Low voltages	3.32 (1.61, 6.85)	***0.001***	3.60 (1.69, 7.67)	***0.001***
Fragmented QRS	1.68 (1.07, 2.62)	***0.02***	2.77 (1.41, 5.46)	***0.003***

CI, confidence interval; LGE, late gadolinium enhancement.

Bold-italic *P*-value are <0.05 indicates statistical significance.

**Figure 2:**
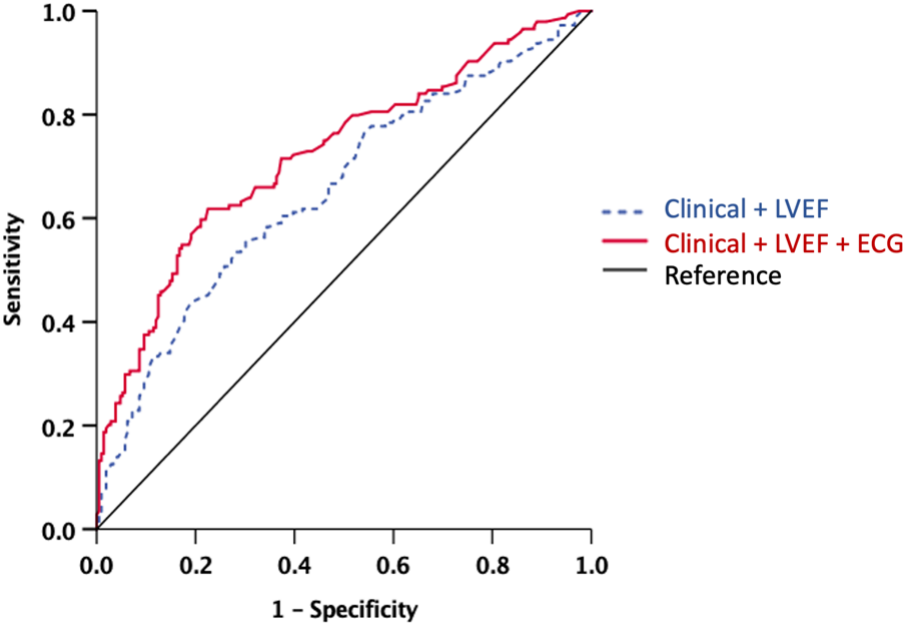
ROC analysis for the presence of LGE. Note the significant increase of the AUC when ECG model was added to the clinical-LVEF model. The AUC for clinical factors + LVEF + ECG was significantly higher than that for clinical factors + LVEF (AUC: 0.66, 95% CI 0.59−0.71 vs. 0.72, 95% CI 0.67−0.78, *p* = 0.003). AUC, area under the curve; ECG, electrocardiography; LVEF, left ventricular ejection fraction; ROC, receiver-operating curve. Clinical model includes systolic blood pressure and history of heart failure. ECG includes lateral inverted T-waves, intraventricular conduction delay, low voltage, and fragmented QRS.

Figure 2 displays the ROC analysis for LGE, comparing clinical factors (systolic BP and history of heart failure, which were associated with LGE in the univariable analysis) + LVEF, and clinical factors + LVEF + ECG (lateral inverted T-waves, IVCD, low voltages, or fragmented QRS). The AUC for clinical factors + LVEF + ECG was significantly higher than that for clinical factors + LVEF (AUC: 0.66, 95% CI 0.59–0.71 vs. 0.72, 95% CI 0.67–0.78, *p* = 0.003).

### Clinical outcomes

**Figure 3:**
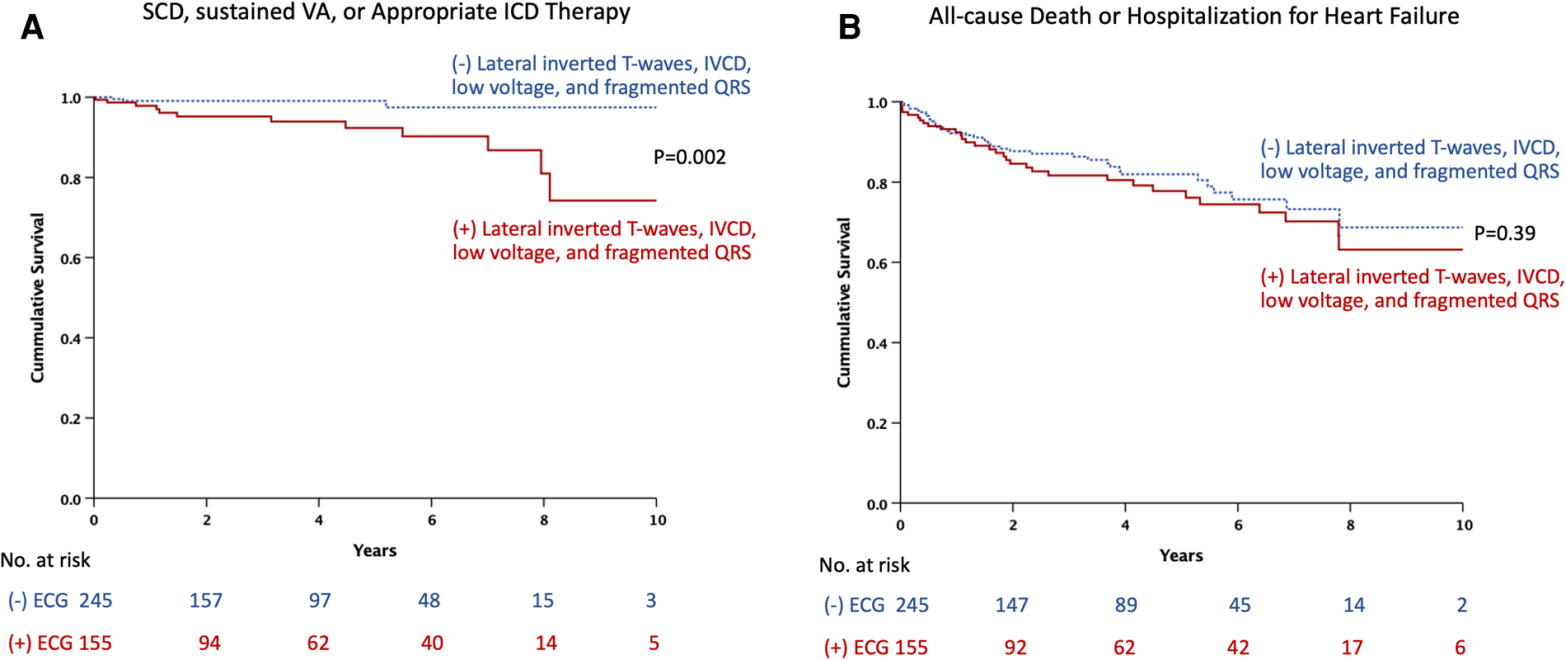
Kaplan-Meier estimates of the time to events by presence or absence of ECG predictors: (**A**) sudden cardiac death, sustained ventricular arrhythmia, or appropriate ICD therapy. (**B**) All-cause death or hospitalization for heart failure. ECG, electrocardiography; ICD, implantable cardioverter-defibrillator; IVCD, intraventricular conduction delay; SCD, sudden cardiac death; VA, ventricular arrhythmia. ECG predictors include lateral inverted T-waves, intraventricular conduction delay, low voltage, and fragmented QRS.

Twenty-two patients did not have follow-up data; therefore, 400 patients were included in the outcome analysis. During a median follow-up period of 2.7 years (IQR 0.8, 5.2), 16 events of SCD, sustained ventricular arrhythmia, or appropriate ICD therapy and 70 events of all-cause death or hospitalization for heart failure occurred. Thirty patients underwent ICD implantation after CMR. Among them, three received appropriate therapy, while three received inappropriate therapy. Table 4 shows the rates of outcomes of patients with and without ECG predictors (lateral inverted T-waves, IVCD, low voltages, or fragmented QRS), respectively. Patients with ECG predictors demonstrated markedly higher rates of SCD, sustained ventricular arrhythmia, or appropriate ICD therapy [8.4% vs. 1.2%, hazard ratio (HR) 5.97, 95% CI 1.69–21.05, *p* = 0.005], while experiencing similar rates of all-cause death or hospitalization for heart failure (20.6% vs. 15.5%, HR 1.22, 95% CI 0.76–1.96, *p* = 0.39). Figure 3 shows Kaplan-Meier curves for composite outcomes in DCM patients, stratified by the presence or absence of ECG predictors.

**Table 4:** Patients outcomes.

	All patients (*n* = 400)	ECG predictors^a^ present (*n* = 155)	ECG predictors^a^ absent (*n* = 245)	Hazard ratio (95% CI)	*P*-value
Sudden cardiac death, sustained ventricular arrhythmia, or appropriate ICD therapy	16 (4.0)	13 (8.4)	3 (1.2)	5.97 (1.69, 21.05)	***0.005***
Sudden cardiac death	3 (0.7)	1 (0.6)	2 (0.8)		
Sustained ventricular arrhythmia	15 (3.8)	13 (8.4)	2 (0.8)		
Appropriate ICD therapy	3 (0.7)	3 (1.9)	0 (0)		
All-cause death or hospitalization for heart failure	70 (17.5)	32 (20.6)	38 (15.5)	1.22 (0.76, 1.96)	0.39
All-cause death	27 (6.8)	12 (7.7)	15 (6.1)		
Hospitalization for heart failure	51 (12.7)	23 (14.8)	28 (11.4)		

CI, confidence interval; DCM, dilated cardiomyopathy; ECG, electrocardiography; ICD, implantable cardioverter-defibrillator.

Bold-italic *P*-value are <0.05 indicates statistical significance.

^a^ ECG predictors include lateral inverted T-waves, intraventricular conduction delay, low voltage, and fragmented QRS.

### Multivariable analyses of composite outcomes

Table 5 demonstrates univariable and multivariable analyses of variables associated with SCD, sustained ventricular arrhythmia, or appropriate ICD therapy. The multivariable analysis revealed that history of sustained ventricular arrhythmia (HR 12.15, 95% CI 4.19–35.21, *p* < 0.001), history of heart failure (HR 5.40, 95% CI 1.20–24.31, *p* = 0.03), LVEDV index (HR 1.01, 95% CI 1.003–1.02, *p* = 0.02), and ECG predictors (HR 4.84, 95% CI 1.34–17.40, *p* = 0.01) were independently associated with SCD, sustained ventricular arrhythmia, or appropriate ICD therapy.

**Table 5:** Univariable and multivariable Cox regression analyses of clinical, electrocardiographic, and CMR predictors associated with sudden cardiac death, sustained ventricular arrhythmia, or appropriate ICD therapy.

	Univariable analysis		Multivariable analysis	
	Hazard ratio (95% CI)	*P*-value	Hazard ratio (95% CI)	*P*-value
Age, per 1 year increment	1.006 (0.97, 1.04)	0.72		*** ***
Male	1.46 (0.50, 4.26)	0.48		*** ***
Body mass index, per kg/m^2^	0.92 (0.83, 1.03)	0.16		*** ***
Systolic blood pressure, per mmHg	1.002 (0.98, 1.02)	0.83		*** ***
Diastolic blood pressure, per mmHg	0.98 (0.95, 1.02)	0.40		*** ***
Chest pain	0.04 (0.00, 297.79)	0.48		*** ***
Dyspnea	1.34 (0.38, 4.71)	0.64		*** ***
Syncope or palpitation	4.30 (1.59, 11.64)	***0.004***		** **
History of heart failure	7.63 (1.72, 33.81)	***0.007***	5.40 (1.20, 24.31)	***0.03***
NYHA functional class, per class	1.41 (0.70, 2.83)	0.33		*** ***
Hypertension	1.12 (0.40, 3.11)	0.81		*** ***
Diabetes mellitus	1.59 (0.59, 4.29)	0.35		*** ***
Hyperlipidemia	1.64 (0.60, 4.42)	0.32		*** ***
Smoker	0.68 (0.09, 5.16)	0.71		*** ***
Alcohol excess^a^	0.60 (0.08, 4.48)	0.62		*** ***
Ischemic stroke	0.04 (0.00, 200.62)	0.46		*** ***
History of sustained ventricular arrhythmia	10.97 (4.05, 29.67)	**<*0.001***	12.15 (4.19, 35.21)	**<*0.001***
ECG				
ECG predictors^b^	5.97 (1.69, 21.05)	***0.005***	4.84 (1.34, 17.40)	***0.01***
CMR				
LVEDV index, per ml/m^2^	1.01 (1.004, 1.02)	***0.008***	1.01 (1.003, 1.02)	***0.02***
LVESV index, per ml/m^2^	1.01 (1.003, 1.02)	***0.01***		*** ***
LV mass index, per g/m^2^	1.01 (0.99, 1.02)	0.28		*** ***
LV ejection fraction, per 1% increment	0.96 (0.92, 1.005)	0.07		*** ***
LGE present	5.77 (1.62, 20.63)	***0.007***		*** ***

CI, confidence interval; CMR, cardiac magnetic resonance; ECG, electrocardiography; LGE, late gadolinium enhancement; LV, left ventricular; LVEDV, left ventricular end-diastolic volume; LVESV, left ventricular end-systolic volume; NYHA, New York Heart Association.

Bold-italic *P*-value are <0.05 indicates statistical significance.

^a^ Defined as consistent intake of 4 or more units/d for men and 3 or more units/d for women.

^b^ ECG predictors include lateral inverted T-waves, intraventricular conduction delay, low voltage, and fragmented QRS.

Table 6 demonstrates univariable and multivariable analyses of variables associated with all-cause death or hospitalization for heart failure. The multivariable analysis revealed that age (HR 1.02, 95% CI 1.005–1.04, *p* = 0.01), NYHA functional class (HR 1.85, 95% CI 1.39–2.48, *p* < 0.001), hyperlipidemia (HR 1.89, 95% CI 1.15–3.08, *p* = 0.01), and LGE (HR 2.10, 95% CI 1.28–3.44, *p* = 0.003) were independently associated with all-cause death or hospitalization for heart failure.

**Table 6:** Univariable and multivariable Cox regression analyses of clinical, electrocardiographic, and CMR predictors associated with all-cause death or hospitalization for heart failure.

	Univariable analysis		Multivariable analysis	
	Hazard ratio (95% CI)	*P*-value	Hazard ratio (95% CI)	*P*-value
Age, per 1 year increment	1.02 (1.005, 1.04)	***0.01***	1.02 (1.005, 1.04)	***0.01***
Male	0.62 (0.39, 1.00)	0.05		*** ***
Body mass index, per kg/m^2^	0.96 (0.91, 1.01)	0.12		*** ***
Systolic blood pressure, per mmHg	1.002 (0.99, 1.01)	0.62		*** ***
Diastolic blood pressure, per mmHg	0.99 (0.98, 1.01)	0.79		*** ***
Chest pain	0.68 (0.25, 1.87)	0.68		*** ***
Dyspnea	1.75 (0.92, 3.34)	0.09		*** ***
Syncope or palpitation	1.28 (0.70, 2.34)	0.42		*** ***
History of heart failure	1.23 (0.77, 1.97)	0.38		*** ***
NYHA functional class, per class	1.92 (1.44, 2.55)	***<0.001***	1.85 (1.39, 2.48)	**<*0.001***
Hypertension	1.55 (0.93, 2.59)	0.09		** **
Diabetes mellitus	1.92 (1.19, 3.06)	***0.007***		** **
Hyperlipidemia	2.20 (1.35, 3.58)	***0.001***	1.89 (1.15, 3.08)	***0.01***
Smoker	1.13 (0.52, 2.49)	0.74		*** ***
Alcohol excess^a^	0.65 (0.24, 1.79)	0.40		*** ***
Ischemic stroke	1.74 (0.83, 3.64)	0.14		*** ***
History of sustained ventricular arrhythmia	1.37 (0.59, 3.16)	0.46		*** ***
ECG				
ECG predictors^b^	1.22 (0.76, 1.96)	0.39		*** ***
CMR				
LVEDV index, per ml/m^2^	1.002 (0.99, 1.008)	0.57		*** ***
LVESV index, per ml/m^2^	1.003 (0.99, 1.008)	0.38		*** ***
LV mass index, per g/m^2^	1.00 (0.99, 1.007)	0.64		*** ***
LV ejection fraction, per 1% increment	0.99 (0.96, 1.008)	0.22		*** ***
LGE present	2.46 (1.52, 3.99)	**<*0.001***	2.10 (1.28, 3.44)	***0.003***

CI, confidence interval; CMR, cardiac magnetic resonance; ECG, electrocardiography; LGE, late gadolinium enhancement; LV, left ventricular; LVEDV, left ventricular end-diastolic volume; LVESV, left ventricular end-systolic volume; NYHA, New York Heart Association.

Bold-italic *P*-value are <0.05 indicates statistical significance.

^a^ Defined as consistent intake of 4 or more units/d for men and 3 or more units/d for women.

^b^ ECG predictors include lateral inverted T-waves, intraventricular conduction delay, low voltage, and fragmented QRS.

## Discussion

The main findings of this study can be summarized as follows: In patients with DCM who underwent CMR, ECG predictors, including lateral inverted T-waves, IVCD, low voltage, and fragmented QRS, were independently associated with LGE. Additionally, ECG predictors added diagnostic value for the identification of LGE, beyond clinical factors and LVEF. Furthermore, ECG predictors were independently associated with an increased risk of SCD, sustained ventricular arrhythmia, or the need for appropriate ICD therapy.

### LGE and cardiovascular events in DCM

LGE CMR is a noninvasive method used to determine the underlying cause of DCM, and previous studies have reported its prognostic value in identifying patients at risk of cardiac events (1–3, 9, 16). Gulati et al. suggested that midwall LGE is independently associated with all-cause mortality, heart failure, as well as SCD (3). Di Marco et al. also reported that LGE is independently associated with SCD or ventricular arrhythmia in a meta-analysis encompassing 2,948 patients with non-ischemic DCM (16). Therefore, LGE can potentially improve risk stratification for cardiac events in patients with DCM. In our study, 40% of DCM patients demonstrated LGE, with the majority exhibiting midwall LGE. The prevalence of LGE in DCM patients varied among studies. Gulati et al. demonstrated that 30% of DCM patients had LGE, focusing solely on midwall LGE (3). Tateishi et al. found that 50.7% of DCM patients had LGE (14). In a meta-analysis conducted by Becker et al., which included 4,554 patients with DCM, LGE was present in 44.8% of cases (4). The prevalence of LGE in our study was comparable to that reported in previous studies. Patients with LGE had a higher prevalence of a history of heart failure and exhibited more severe degrees of LV remodeling, including higher LV volumes and lower LVEF.

Patients with LGE demonstrated significantly higher rates of all-cause death and heart failure hospitalization. These findings are consistent with prior studies (2–4). Patients with LGE were also associated with SCD and sustained ventricular arrhythmia in the univariable analysis, but this was not shown in multivariable analysis. However, given the relatively low number of events, caution should be exercised when interpreting these findings.

### Electrocardiography as a predictor of LGE

The majority of patients with DCM exhibit abnormal ECG findings, including cardiac chamber enlargement, bundle branch block, and inverted T-waves. In our study only 10% had normal ECG, which was consistent with a study of Merlo (7). ECG findings in DCM can result from either abnormal cardiac structure or genetic factors associated with DCM (9). Our study was the first to demonstrate ECG characteristics associated with LGE including lateral inverted T-waves, IVCD, low voltage, and fragmented QRS. The reduction in QRS amplitude, particularly in the precordial leads, can be attributed to the loss of vital myocardium and diffuse LV fibrosis (7, 17, 18). In cases of DCM caused by muscular dystrophies, posterior or inferior Q waves often manifest, reflecting transmural myocardial fibrosis (19). Inverted T-waves, especially in the lateral leads, is a recognized feature of certain genetic forms (such as filamin C or desmosomal disease) (20). DCM resulting from prior myocarditis presents a wide spectrum of non-specific ECG findings, including low voltages, conduction abnormalities, lateral inverted T-waves, increased QRS duration, and PVC/nonsustained ventricular tachycardia (4). Although we did not have genetic data to prove an association between ECG findings and specific genetic factors, our findings contribute ECG predictors of LGE, potentially aiding clinicians in selecting patients with DCM for CMR when its availability is limited, particularly in developing countries. We also believe that certain ECG findings could provide additional prognostic value, complemented by benefits from clinical and CMR assessments.

### Prognostic value of electrocardiography in DCM

Several studies have demonstrated the prognostic value of ECG in patients with DCM. Merlo et al. demonstrated that anterolateral inverted T-waves predicted study outcomes, including death or heart transplant, as well as sudden death or malignant ventricular arrhythmias (7). A CMR study by Marume et al. showed that the combination of LGE and a wide QRS complex provided additional prognostic stratification compared to LGE status alone, potentially enhancing the appropriate use of ICD therapy in patients with DCM (21). Low QRS amplitude and fragmented QRS complex also carry a heightened risk for major ventricular arrhythmias and adverse cardiac events (6, 8, 9). Our study demonstrated that ECG predictors were independently associated with SCD, sustained ventricular arrhythmia, or appropriate ICD therapy. This is largely consistent with the aforementioned studies. Lateral inverted T-waves were one of the ECG characteristics that were consistently associated with SCD and malignant ventricular arrhythmia in both our study and the study by Merlo (7). The mechanism behind this could be related to DCM caused by post-inflammatory processes. Another mechanism could arise from specific genotypes, such as intercellular junction protein mutations, which underlie possible overlapping phenotypes between DCM and arrhythmogenic cardiomyopathy. Consequently, the overall prognostic role of lateral inverted T-waves was mainly driven by their association with major arrhythmic events (4, 7, 22). In our study, ECG predictors were not associated with all-cause death or hospitalization for heart failure. This could be explained by several reasons, such as ECG findings like atrial fibrillation or left atrial enlargement may be consequences of severe heart failure rather than predictive factors.

### Study limitations

This study had some limitations. Firstly, single-time ECG data may change dynamically during a follow-up period. However, using a single ECG is practical for clinicians to assess. Secondly, the study population was limited to a CMR center within a tertiary hospital, introducing selection bias, and the characteristics of the patients analyzed may differ from those of the general population with DCM. Thirdly, we have not included the impact of different LGE patterns in our analysis, as the majority of patients (>70%) exhibited midwall LGE. Other types of LGE could represent specific cardiomyopathies such as burnout hypertrophic cardiomyopathy or varying degrees of hypertensive heart disease. However, the prevalence of these populations was low, reflecting real-world data that are consistent with prior published research. Lastly, we did not have T1 mapping and extracellular volume fraction data that had prognostic value in patients with DCM (23, 24). However, there is little evidence of incremental value when LGE is already a routine part of the scanning protocol (24, 25).

## Conclusions

In patients with DCM, lateral inverted T-waves, IVCD, low voltage, and fragmented QRS were independently associated with LGE. Additionally, these ECG predictors had prognostic value for predicting SCD, sustained ventricular arrhythmia, or appropriate ICD therapy, assisting clinicians in stratifying SCD risk and identifying primary prevention ICD implantation candidates.

## Data Availability

The raw data supporting the conclusions of this article will be made available by the authors, without undue reservation.
