# Supplementary material for: Electrocardiographic characteristics associated with late gadolinium enhancement and prognostic value in patients with dilated cardiomyopathy
Source: Front Cardiovasc Med. 2023 Oct 18;10:1281563. doi: 10.3389/fcvm.2023.1281563 (PMC10619146; doi:10.3389/fcvm.2023.1281563)
Supplement: Supplementary file 1 [file Table1.docx]

**Supplemental Materials**

**Supplemental Table 1.** Univariable Cox Regression Analysis of Clinical and Electrocardiographic Predictors Associated with LGE.

|  | Univariable Analysis | |
| --- | --- | --- |
|  | Odd Ratio (95% CI) | P-value |
| Age, per 1 year increment  Male  Body mass index, per kg/m^2^  Systolic blood pressure, per mmHg  Diastolic blood pressure, per mmHg  Chest pain  Dyspnea  Syncope or palpitation  History of heart failure  NYHA functional class, per class  Hypertension  Diabetes mellitus  Hyperlipidemia  Smoker  Alcohol excess  Ischemic stroke  Sustained ventricular arrhythmia | 0.99 (0.98, 1.01)  1.30 (0.88, 1.94)  0.97 (0.94, 1.01)  0.98 (0.97, 0.99)  0.99 (0.98, 1.01)  0.77 (0.36, 1.64)  1.55 (0.97, 2.47)  1.59 (0.89, 2.84)  1.90 (1.28, 2.82)  1.32 (0.99, 1.74)  0.72 (0.48, 1.06)  1.07 (0.71, 1.62)  1.28 (0.86, 1.91)  0.98 (0.51, 1.86)  0.97 (0.49, 1.93)  0.99 (0.47, 2.13)  1.68 (0.72, 3.90) | 0.41  0.19  0.15  ***0.002***  0.30  0.49  0.07  0.12  ***0.001***  0.05  0.09  0.74  0.21  0.94  0.94  0.99  0.23 |
| **ECG**  Normal ECG  Heart rate, per 1 beat per minute increment  PR interval, per 1 ms  QRS duration, per 1 ms  QRS complex widening  QT interval, per 1 ms  Left atrial enlargement  Right atrial enlargement  Left ventricular hypertrophy (Sokolow-Lyon)  Left ventricular hypertrophy (Cornell)  Right ventricular hypertrophy  Anterior inverted T-waves  Inferior inverted T-waves  Lateral inverted T-waves  Anterior ST depression  Inferior ST depression  Lateral ST depression  Anterior Q waves  Inferior Q waves  Lateral Q waves  First-degree atrioventricular block  Premature ventricular complex  Left bundle branch block  Complete right bundle branch block  Intraventricular conduction delay  Low voltage  Fragmented QRS  Wolff-Parkinson-White syndrome | 0.70 (0.35, 1.36)  0.99 (0.98, 1.01)  1.004 (0.99, 1.01)  1.007 (1.00, 1.01)  1.40 (0.91, 2.16)  1.001 (0.99, 1.005)  1.23 (0.77, 1.96)  1.07 (0.33, 3.43)  0.64 (0.39, 1.05)  1.02 (0.60, 1.72)  0.59 (0.11, 3.09)  2.96 (0.53, 16.36)  4.42 (0.46, 42.98)  5.46 (1.97, 15.17)  1.77 (0.53, 5.93)  2.47 (0.58, 10.50)  1.62 (0.79, 3.31)  2.72 (0.89, 8.29)  2.62 (0.75, 9.12)  1.77 (0.53, 5.92)  1.86 (0.95, 3.67)  1.34 (0.79, 2.29)  0.86 (0.50, 1.48)  1.40 (0.60, 3.25)  3.42 (1.44, 8.13)  3.32 (1.61, 6.85)  1.68 (1.07, 2.62)  0.50 (0.05, 4.81) | 0.29  0.84  0.23  0.06  0.13  0.56  0.39  0.91  0.08  0.96  0.53  0.21  0.20  ***0.001***  0.35  0.22  0.19  0.08  0.13  0.35  0.07  0.28  0.58  0.43  ***0.005***  ***0.001***  ***0.02***  0.55 |

A *p*-value<0.05 indicates statistical significance.

**Abbreviations:** CI, confidence interval; ECG, electrocardiography; LGE, late gadolinium enhancement; NYHA, New York Heart Association.
